# Migration as a Determinant in the Development of Children Emotional and Behavior Problems: A Quantitative Study for Lisbon Region, Portugal

**DOI:** 10.3390/ijerph18020375

**Published:** 2021-01-06

**Authors:** Zélia Muggli, Thierry Mertens, Silva -Sá, Regina Amado, Ana L. Teixeira, Dora Vaz, Maria Rosário O. Martins

**Affiliations:** 1 Global Health and Tropical Medicine, Institute of Hygiene and Tropical Medicine, NOVA University of Lisboa, 1349-008 Lisbon, Portugal; zelia.muggli@ihmt.unl.pt (Z.M.); thierry.mertens@ihmt.unl.pt (T.M.); a21000990@ihmt.unl.pt (S.-S.); regina.amado@ihmt.unl.pt (R.A.); 2 Interdisciplinary Centre of Social Sciences (CICS.NOVA), Faculty of Social Sciences and Humanities (NOVA FCSH), 1070-312 Lisbon, Portugal; analuciateixeira@fcsh.unl.pt; 3 Amadora Primary Care Health Centers Group, Regional Health Administration of Lisbon and Tagus Valley, 2700-856 Amadora, Portugal; dora.vaz@arslvt.min-saude.pt

**Keywords:** child mental health, migration, social determinants, internalizing behaviors, externalizing behaviors, strengths and difficulties questionnaire

## Abstract

The role of migration as a determinant in child mental health has been demonstrated in a number of studies. However, results are not always consistent, and the research continues to be scarce, especially in Portugal. We examined the association between sociodemographic profiles and the chance for the development of emotional and behavioral difficulties in a group of 420 children, immigrant (*n* = 217) and born in Portugal to Portuguese born parents (*n* = 203). We used a structured questionnaire to obtain sociodemographic information and the Strength and Difficulties Questionnaire (SDQ). Descriptive statistics were used to characterize children and their families; variables were compared between groups using the Chi-squared, Fisher’s Exact Test, or the Mann–Whitney U test and logistic regression was used to analyze the association between socio-demographic factors and emotional and behavioral difficulties. Results showed a pattern of social and mental health inequalities with immigrant children at a disadvantage: they are more often part of families with low income and where parents had low skilled jobs. Internalizing behaviors are more frequent in immigrants than in children born in Portugal to Portuguese-born parents (*p* = 0.001) whereas a high total SDQ difficulties score (*p* = 0.039) and externalizing behaviors were more frequent in 1st generation immigrant children (*p* = 0.009). A low family income (aOR 4.5; 95% CI: 1.43–13.95), low parental education level (aOR 2.5; 95% CI: 1.11–5.16), and being a first-generation immigrant child (aOR 2.2; 95% CI: 1.06–4.76) increased significantly the chance of developing emotional and behavioral difficulties. This study contributes to the identification of children vulnerable to mental health problems who can benefit from monitoring, early detection and preventive interventions in order to mitigate possible negative outcomes in the future.

## 1. Introduction

Several studies estimated that emotional, developmental, and behavior problems may affect 10–20% of children globally [1], being the main cause of disability in young people. If left unresolved, the development of mental health problems in childhood can have long-lasting effects on overall development and attainments, having an immediate effect on the child’s life at school, home, and community as well. Moreover, these children often endure challenges with stigma and lack of widely available access to mental healthcare services [1]. Early childhood is a key period in researching determinants for eventual mental health problems. It is defined as the period from prenatal development to eight years of age, encompassing the most crucial stages to growth, health, and development throughout the life course [2]. At the same time, it is the period most influenceable by external factors and a phase of increased vulnerability. Adverse effects resulting from the complex interaction of environmental, social, and biological influences during this period have the potential to be irreversible [3]. Consequently, policies aiming at improving health and wellbeing outcomes in early childhood may have a lasting impact and higher returns than later inputs.

Effects on children’s mental health have been attributed to a number of factors which may interact with each other. Preterm and low birthweight births have been related to disadvantageous outcomes [4,5,6,7,8], although other studies have implied that these effects may be modified by socioeconomic status [9]. A longer duration of breastfeeding has been linked to less likelihood of developing emotional and behavioral difficulties [10]. Family structure can have an impact on children’s sociopsychological development. Children who live with both parents appear to have less emotional and behavioral problems. Conversely, children in single-parent families, who often have more economic insecurity and where a single career may face added stress and loneliness, seem to encounter multiple emotional and behavioral challenges [11,12]. However, the main body of research points to a low socioeconomic status as a key determinant for the development of mental health problems in children.

Over the last years, the role of migration as a health determinant has drawn more attention as a growing number of the population in Europe is made up by immigrants. The pathways through which health can be affected by the migratory process are complex. They start in the country of origin including the individual’s own health and family’s health behaviors before departure, local patterns of disease, cultural practices, and access to health services [13]. The conditions individuals face when traveling and the interactions with the new socioeconomic and cultural context in the country of destination require responses and adaptations that can make migration a stressful event [14]. Many factors in this process can negatively influence a child’s mental health. They include differences in the family and host country societal values, adaptation to a new language, asymmetric acculturation within families—referring to when children acquire the host country culture faster than the parents—and frequent exposure to discrimination and low socioeconomic status [15,16]. Additionally, a non–European origin, younger age at immigration, gender effects, and maternal harsh parenting style have been also suggested as major influences [16,17]. Yet, as this process can be experienced in diverse ways, the impact it can have differs. A systematic review of 36 studies in European countries conveyed immigration as a risk factor for mental health problems, especially in the case of 1st generation immigrant children with the most disadvantaged outcomes having been found in refugee children [18,19,20].

Emotional and behavioral difficulties in childhood may be summarized in two groups based on symptoms: internalizing problems—consisting of depression, anxiety, and somatic manifestations and externalizing problems—entailing hyperactivity, aggressiveness, and defiant behaviors [21]. Internalizing problems have been found more frequently in immigrant children, whereas externalizing problems do not seem to differ between immigrant and non-immigrant children, except for adolescent immigrants that have been living longer in the country of destination [22]. Although many studies support a migrant background as an increased risk to develop emotional and behavioral problems in children, a smaller number report minimal or no differences between immigrant children and their non-immigrant counterparts [23] or even an advantage for immigrant children [24,25]. This inconsistency of findings might result from the heterogenicity of the immigrant groups among countries or even among regions in a country. Therefore, the relevance of conducting research at a more regional level within a single country.

In Portugal, the immigrant population has seen an increase in size. The global financial crisis of 2008 brought a decrease in the number of foreign residents, but from 2016 the numbers increased annually to a total of 480.300 registered foreign residents in 2018, the highest number ever registered. The biggest community comes from Brazil (21.9%), followed by Cape Verde (7.2%), but more diverse origins were observed, mainly from Asia, including an increase of over 100% in the Bengali, Nepalese, and Indian communities compared with the previous year [26]. In spite of the recent increase in numbers and the increasingly diverse nature of the immigration influxes in Portugal, the knowledge base on the effects of immigration on child health, and in particular, their expression in the mental health aspect, is scarce. This field of research has been identified as a priority by several authors [13,27]. A better understanding of the social and mental health profiles of children can contribute to policies aimed at multisectoral prevention, early identification, and interventions, in order to help them reach their full potential when starting school and to shape better future health and wellbeing outcomes.

The objectives of this paper are to explore the sociodemographic profile and emotional and behavioral difficulties of 4-year-olds, comparing immigrant children and children born in Portugal to Portuguese parents in the metropolitan area of Lisbon, Portugal, and to analyze factors associated with the likelihood of developing those difficulties.

## 2. Materials and Methods

### 2.1. Study Population and Participants 

We utilized data from a cohort of children born during 2015 and living in Amadora, Metropolitan Area of Lisbon, Portugal. The cohort study aims to analyze children born in Portugal to Portuguese-born parents and immigrant children health trajectories, including physical and psychomotor development, emotional and behavioral problems, utilization of health services, and their sociodemographic contexts. The present paper used the first cohort wave data set.

Amadora is the most densely populated municipality in the country and the fourth most populous city in Portugal; moreover, 10% of its population had a foreign nationality, namely from Portuguese speaking countries (Brazil, Cape-Verde, Angola, and Guinea-Bissau).

To be eligible, children needed to be born in 2015 and attending one of the 9 primary health care centers (PHCC) consultations; 1009 children were eligible for which we collected clinical data based on medical records. If we assumed that 30% of them are immigrants (data from previous study) our target population comprised 302 immigrant children. In order to compare health outcomes by immigrant status, we considered also 302 born in Portugal to Portuguese born parents children with a study population of 604 children. We randomly select the weeks of recruitment in each health center; then for each week and each day we selected children that were available for the consultations until the sample size for the corresponding health centers was attained. To obtain additional information, namely on demographic and socioeconomic information, migration status, and children’s emotional and behavior problems we surveyed parents/caregivers of children between June 2019 and the first week of March 2020, during routine health assessments or other appointments. Recruitment weeks were distributed randomly among the PHCC and the number of children recruited was proportional to the dimension of each health center.

### 2.2. Instruments

Two questionnaires were applied: the first was a pilot-tested structured questionnaire, administered by face-to-face interview to obtain information on the sociodemographic characteristics and immigrant history of the children and parents/caregivers; the second was the Strengths and Difficulties Questionnaire (SDQ), self-administered to parents, for 4–17 years old in the Portuguese validated parent’s version by Fleitlich, Loureiro, Fonseca, and Gaspar [28] or in the parents’ language when these did not speak Portuguese. The SDQ was only administered to one parent or caregiver accompanying the four-year-old child to the PHCC. The SDQ is a brief questionnaire to assess child emotional and behavioral difficulties, with psychometric properties [29,30], namely on 4-year-olds. It is available in over 60 languages and has been widely used and validated in various research, clinical, and community settings, including in a multi-ethnic population of children [19,31] and in several countries, such as Portugal [32]. The SDQ can be completed by parents (SDQ 2–4 and 4–17 years), teachers (SDQ 4–17 years), and the children themselves in a self-reported version (11–17 years). It consists of 25 items relating to children’s strengths and difficulties, configured in 5 subscales, 4 of which measure difficulties: emotional symptoms, conduct problems, hyperactivity-inattention, peer problems, and 1 subscale measuring a strength: prosocial behavior. Each subscale is assessed with 5 questions, related to symptoms perceived over the last six months, answered with a three-point Likert-type scale “not true,” “somewhat true,” or “certainly true.” The 4 difficulties subscales can be summarized in 2 broader externalizing (sum of conduct problems and hyperactivity subscales) and internalizing (sum of emotional and peer problems subscales) behaviors; this configuration has been proposed for use in epidemiological studies and in low-risk populations.

A team of 6 interviewers, junior researchers with origin in 5 different Portuguese speaking countries, recruited the parents/caregivers and conducted the interviews in especially allocated rooms in the PHCC to assure privacy. To address intra and inter-reliability in interviews, a structured questionnaire was used, variables and their categorization were clearly defined, and all interviewers received the same detailed information and extensive training on the interview process. The main language used was Portuguese, but in some cases other languages such as Creole and English were necessary.

### 2.3. Measures

Immigrant status was the main explanatory variable of interest together with other socioeconomic and demographic factors related to both children and parents/caregivers. Immigrant status was defined at two levels: first and second generation, together with the country of birth and language spoken at home, and length of stay in Portugal (in months) for 1st generation immigrant children. We included several other variables such as birth weight (<2500 g–low birthweight, ≥2500 g); gestational age (<37 weeks–preterm, ≥37 weeks); breastfeeding (yes/no) and its duration in months, as literature suggests they can have potential effects on the development of emotional and behavioral difficulties [4,5,6,7,8,10]

We measured the main outcome of interest (SDQ) at three levels: using the five subscales, where the score for each subscale can go from 0 to 10 points categorized in normal, borderline and abnormal; the SDQ Total Difficulties Score, calculated by adding up the scores for the emotional problems, hyperactivity, conduct problems, and peer problems, with values between 0–40, also categorized in 3-bands, where 0–13 points = normal, 14–16 points = borderline, 17–40 = abnormal; using internalizing (sum of emotional and peer problems subscales) and externalizing (sum of conduct problems and hyperactivity subscales) problems scores, which can have values 0–20 where higher values indicate bigger difficulties; and finally we dichotomize the SDQ Total score in a binary variable indicating if a child has emotional and behavioral difficulties in general (score ≥ 14) or not (score 0–13 considered “normal”).

### 2.4. Statistical Analysis

Descriptive statistics were used to characterize children and their families; we disaggregated data into two groups: born in Portugal to Portuguese born parents and immigrant children as defined above. To analyze children’s emotional and behavioral difficulties, we computed frequencies of the 3 categories (normal, borderline, abnormal) for the five SDQ subscales and for the Total of Difficulties Scores and compared them between the groups of interest. Externalizing and internalizing behaviors were explored using the quantitative scores corresponding to each behavior. To compare categorical variables between groups we used the Chi-squared association test or Fisher’s Exact Test while for quantitative variables we used the Mann–Whitney U test.

To analyze factors associated with the chance of developing emotional and behavioral difficulties, we estimated logistic regression models with “emotional and behavioral difficulties in general” binary score as a dependent variable. Adjusted odds-ratios together with 95% CI were calculated. Data analysis was conducted using the software Statistical Package for the Social Sciences (SPSS^®®^) version 26.0 (IBM Corp., Armonk, NY, USA). A 5% significance level was used in statistical analysis.

### 2.5. Ethical Considerations

The project was reviewed and approved by the Research Ethics Committee of the Regional Health Administration of Lisbon and Tagus Valley, Portugal (001/CES/INV/2019). All participating parents/caregivers gave their written informed consent before inclusion in the study.

## 3. Results

### 3.1. Children and Parent’s/Caregivers Characteristics

We collected questionnaires information on 203 children born in Portugal to Portuguese born parents and 217 immigrants (51.7%), of which 41 are 1st generation (9.8%), with a response rate of 70% (*n* = 420). The 41 children born outside of the EU came mostly from the Community of Portuguese Speaking Countries (CPLP): 13 from Brazil, 8 from Angola, followed by Guinea-Bissau with 6.

Children from countries less traditionally associated with immigration to Portugal, such as India, Nepal, or Eritrea, are also present in the study. The median length of stay of children in Portugal was 18 months (1 min.—48 max.). Portuguese was the only language spoken in 268 households (64%). Other 17 languages were spoken, ranging from Nepalese to Mandarin and Tigrinya. After the Portuguese, the most common language spoken was a combination of Creole and Portuguese (20%), spoken not only by immigrant families but also in 6.4% of households of children born in Portugal to Portuguese born parents. The main sociodemographic and biological characteristics of the children are summarized in Table 1 and Table 2.

No differences were found among the groups for gestational age, birthweight, and having been breastfed. However, the duration of breastfeeding was significantly higher (*p* < 0.001) for 1st generation immigrant children with a median of 18 months against 6 months for children born in Portugal to Portuguese born parents and 12 months in immigrant children in general. While no differences were found in family structure between groups, childcare arrangements in 1st generation immigrant children were different from the other groups (*p* < 0.001) with 58.5% attending pre-school versus 80–85% in the other groups.

The main sociodemographic characteristics of the parents/caregivers are listed in Table 3. Questionnaires respondents were 87.6% women with a median age of 35. There was no significant difference in the distribution of educational level between groups. Results showed that more specialized jobs were observed in Portuguese families and non-qualified low skilled workers were mostly from families of immigrant background (*p* < 0.001). Unemployment and precarious jobs were more frequent in immigrant families. The distribution of family income was significantly different between groups with more than three times (17.6% versus 4.4%) the families of children born in Portugal to Portuguese born parents receiving above 2000 €/month, and nearly three times (18.5% versus 6.7%) more immigrant families having an income of less than 500 €/month (*p* < 0.001). The main reasons for immigration given by the mother (*n* = 152) were reported to be family reunification (28.9%), obtaining a better education (27.6%), economic reasons (22.4%), with 3.3% having moved because of war.

### 3.2. Strengths and Difficulties Questionnaire Results

The main results for the SDQ are presented in Table 4 and Table 5 and suggested that 35% of the children had emotional or behavioral problems in general.

Looking into the subscales, three areas had a more positive outcome, with over 70% of the children with reported scores within the normal limits: pro-social score, peer problems, and emotional problems. Immigrant children presented a higher occurrence of emotional problems than children born in Portugal to Portuguese born parents (*p* = 0.002). Furthermore, first generation immigration children scores for conduct problems (*p* = 0.039), hyperactivity (*p* = 0.008), and the total SDQ score (*p* = 0.039) were more often above the normal and borderline limits than those of children born in Portugal. 

There was a difference in median internalizing behaviors score between immigrant and children born in Portugal to Portuguese born parents (*p* = 0.001), with immigrant children presenting more internalizing behaviors. Furthermore, the median score was different between 1st generation immigrant children and children born in Portugal (p = 0.009) for externalizing behaviors, with these being more present in 1st generation immigrant children.

### 3.3. Factors Associated with the Chance of Developing Emotional and Behavioural Problems

Two logistic regression models were estimated to identify factors associated with the likelihood of a child to develop emotional and behavioral difficulties. The dependent variable is the SDQ Total score dichotomized; we construct a binary variable indicating if a child has emotional and behavioral difficulties in general (YES if score ≥14) or not (NO if score 0–13, i.e., considered “normal”).

The explanatory variables used in both models were gender of the child, gestational age, household monthly income, and education of the parent/caregiver; the first model used the variable “immigrant child”—child born outside the EU or born in Portugal to mother and/or father born outside the EU, with reference category—children born in Portugal to Portuguese born parents, while the second model used “1st generation immigrant child”—child born outside the EU, instead, with reference category—children born in Portugal. Results are presented in Table 6 and Table 7.

Estimated coefficients from the logistic regression are at the basis of computing the adjusted odds ratio (aOR), an association measure very popular in epidemiology; for example, an adjusted odds ratio greater than one means that the corresponding variable is a risk factor for the probability of developing emotional and behavior problems, adjusting for the other variables of the model. Based on this interpretation, we can see that variables gender and gestational age, in both models, were not associated with the chance of developing emotional and behavioral problems because the corresponding aOR is close to one (*p* value greater than 5%). While in model 1 being an immigrant child is not associated with the chance of developing emotional and behavior problems (aOR = 1), in model 2 the aOR > 1, meaning that being a 1st generation children is a risk factor; in other words, a 1st generation immigrant child is 2.2 times more likely to develop emotional and behavioral problems than a child born in Portugal (95% CI: 1.062–4.756), adjusting for other variables. Moreover, a child in a family having an income of less than 500 €/month is 4.5 times more likely to develop emotional and/or behavioral difficulties than a child in a family with an income of more than 2000 €/month; in addition, the odds of developing these difficulties increases as parent’s educational level decreases.

## 4. Discussion

Considering that research migrant child health and its determinants have been identified as a global priority [13], particularly regarding the mental health aspects, the objectives of this study were to describe and compare the sociodemographic profile and emotional/behavioral difficulties among immigrant and children born in Portugal to Portuguese born parents in Amadora (Metropolitan Area of Lisbon) and to identify factors associated with the chance of developing such difficulties. Our results are important as they act as pathfinders because little is known about child mental health in Portugal, in particular among immigrant children.

Furthermore, it should still be possible to formulate multisectoral interventions for children at this crucial age in the hope to influence positively future health outcomes.

More than half of the 420 children in our study had an immigrant background and around 10% were first generation (born outside the EU). This fact highlights the important role of the Amadora PHCC in the provision of healthcare to this population and the need for a culturally sensitive approach, combined with sufficient resources to allocate a named family doctor to all families, giving special attention to recent arrivals who often lack this allocation. In our study, the main countries of origin for the 1st generation immigrant children were from the CPLP—Brazil (31.7%), Angola (19.5%), Guinea-Bissau (14.6%), and Cape Verde (4.8%). Among the second-generation sample of immigrant children, parents born outside Portugal were mostly from Cape Verde (33.8%), followed by Angola (15.8%), Brazil (15.8%), and Guinea-Bissau (12%), 6% were Non-European from a Non-CPLP country.

Countries less traditionally linked to immigration to Portugal, such as India, Nepal, or Eritrea were also present in the study, attesting to the recent increased heterogeneity of the immigrant population. Nearly 7% of children born in Portugal to Portuguese born parents were from families whose language at home was a combination of Creole and Portuguese, possibly indicating a further immigration background.

The fact that 30% of children lived in extended families in all groups can be related with the combination of resources to face a lower socioeconomic status. This can have implications when developing and implementing health messages or interventions, as intergenerational perceptions among family members may differ. Pre-school was attended by 85% of all children, with slightly more children in the private sector, while 1st generation immigrant children had different childcare care arrangements with 40% not attending pre-school. Both findings might signal insufficient provision of fee-free childcare places.

Respondents consisted of women in 87.6% of the cases, suggesting that fathers or male caregivers have a lesser role in accompanying and making the decision to take the child to health appointments. Although the educational level did not differ between the groups, there are differences in types of occupation and household income disparities between families of immigrant and families of children born in Portugal to Portuguese born parents. Nearly 20% of immigrant families had an income below 500€/month. In line with previous research, specialized and highly skilled jobs were more frequent in the families of children born in Portugal to Portuguese born parents, whereas the non-qualified low skilled occupations were found mostly in families of immigrant children. Lower income in immigrant families is a repeated pattern found in all geographies [15,16]. Regarding emotional and behavioral difficulties, 65.4% of all children had no reported difficulties in the overall score, yet immigrant children had a higher frequency of reported emotional difficulties (35%) and more internalizing problems. Overall difficulties as measured by the SDQ Total Difficulties Score, conduct and hyperactivity subscales scores were higher in 1st generation immigrant children who also presented more externalizing behaviors. These results are consistent with extensive literature [17,22] showing migration, especially in first generation immigrant children, as a determinant for developing emotional and behavioral difficulties. However, the proportion of children with borderline and abnormal scores for the hyperactivity and conduct scores among first generation immigrant children was around 50% and 60% respectively. This needs to be interpreted with caution. Some studies have shown the Total Difficulties SDQ scores to be a more reliable indication of psychosocial functioning in children this age than the subscales [33], including in a multi-ethnic group [34]. Furthermore, the way parents report about their children might be affected by a different culture in their country of origin as a result of tendencies in response style, different expectations, and desirability of a child’s behavior. No study has yet performed this evaluation in Portugal, nevertheless, research has shown the SDQ continues to be a valid instrument in diverse settings [31,34].

While immigrant status was not associated with increased odds of developing emotional and behavioral difficulties, 1st generation immigrant children have a chance 2.2 times higher than children born in Portugal to develop such difficulties; this could be a result of the migration process, including adjustment to the new country where they often live in disadvantaged environments. Our results also confirm the known relationship between economic deprivation and a child’s mental health that has been widely established in research [35,36,37]: adjusting for other factors, for children in our study, as household monthly income decreases, the odds of developing emotional and behavioral difficulties significantly increases. Additionally, the chance of developing difficulties increases as parents’ level of education decreases. A very similar result has been described in the literature related to parental low educational level and the risk for attention deficit hyperactivity disorder or depression [38,39,40].

This study has potential limitations. Although these children are enrolled in a cohort study, the investigation in this paper is based on first wave data (one period of time) and as such, causal relationships may be difficult to derive. Only families which are users of the Amadora PHCC were enrolled. Thus, it would be important to understand if children non PHCC users, especially those with a migration background, have similar factors associated with the probability to develop emotional and behavioral problems. The SDQ data was reported only by one informant, in most cases the mother. As mentioned previously these children are enrolled in a cohort study and the longer-term effects can be followed, but in order to have a better understanding of the psychosocial functioning of the child in different settings, data collection at school age could include the SDQ teacher version as well.

The SDQ psychometric properties have been positively evaluated in community samples in Portugal [32,41] However, a wider multicultural dimension could be included in future research to look further into normative data and cut off points. Carrying out a comparative study on the emotional and behavior problems of children with the same age in the country of origin of the first generation immigrant children could help to better understand changes during the migration process.

It is also essential to scale up information systems at regional and local levels that can look at health indicators, including by migration background, especially in settings with a high immigrant population. At the same time, it is a priority to establish widely accessible specialized child mental health and psychology services close to PHCC, within a framework that at present needs to expand its limited resources. This might be as relevant as ever given the generalized current socioeconomic context with growing inequalities and poverty, and its likely consequences on the mental health of children.

## 5. Conclusions

The results of this study underscore the importance of family income, parental education level, and migration background as “structural” determinants for the chance of developing emotional and behavioral difficulties in four-year-old children. Social and mental health inequalities were present at this early stage of the life cycle with the potential to impact present and future outcomes in health and wellbeing. We identified first-generation immigrant children as more vulnerable to mental health problems.

This combination of factors highlights the need to promote early identification and formulate, together with all interested parties, multisectoral and participatory interventions to reduce mental health problems in the adult age. Primary health care centers are in a privileged position to take part in this task, together with the schools, as they are very familiar with the families and communities’ needs and resources. Multidisciplinary groups, including the school health teams, local authorities, together with the social and economic sector, endowed with adequate resources and in partnership with community stakeholders, need to articulate prevention and socio-economic strategies to ensure that 4 years-old children are better equipped to pursue a harmonious future.

## Figures and Tables

**Table 1:** Characteristics of the immigrant children versus children born in Portugal to Portuguese-born parents.

	Total	Immigrant Children	Children Born in Portugal to Portuguese Born Parents	Statistical Test Value	*p* Value
**Variables**	n (%)	n (%)	n (%)		
	420 (100)	217 (51.7)	203 (48.3)		
**Gender**				Pearson Chi square	
Girls	207 (49.3)	109 (50.2)	98 (48.3)	0.16	*p* = 0.689
Boys	213 (50.7)	108 (49.8)	105 (51.7)		
**Gestational age**				Pearson Chi square	
<37weeks—Preterm	33 (8)	15 (7)	18 (9)	0.538	*p* = 0.463
>37 weeks	380 (92)	198 (93)	182 (91)		
**Birthweight**				Pearson Chi square	
<2500 g—Low Birth Weight	35 (9)	19 (9.9)	16 (8.1)	0.394	*p* = 0.531
>2500 g	353 (91)	172 (90.1)	181 (91.9)		
**Breastfeeding**				Pearson Chi square	
Yes	382 (91.2)	202 (93.1)	180 (89.1)	2.057	*p* = 0.152
No	37 (8.8)	15 (6.9)	22 (10.9)		
**Childcare**				Pearson Chi square	
State pre-school	164 (39.1)	83 (38.4)	81 (39.9)	3.438	*p* = 0.329
Private pre-school	192 (45.8)	94 (43.5)	98 (48.3)		
Stays home w/mother	23 (5.5)	15 (6.9)	8 (3.9)		
Other	40 (9.5)	24 (11.1)	16 (7.9)		
**Duration of breastfeeding**				Mann–Whitney U	
Median in months (min-max)	10 (0–53)	12 (0–53)	6 (0–48)	3927.5	*p* = 0.000
**Family structure**				Pearson Chi square	
Both parents	216 (51.6)	99 (45.8)	117 (57.6)	6.640	*p* = 0.084
Both parents and others	57 (13.6)	30 (13.9)	27 (13.3)		
Single-parent families	70 (16.7)	42 (19.4)	28 (13.8)		
One parent and others/others	76 (18.1)	45 (20.9)	31 (15.3)		

**Table 2:** Characteristics of the first generation immigrant children versus children born in Portugal.

	Total	First Generation Immigrant Children	Children Born in Portugal	Statistical Test Value	*p* Value
**Variables**	n (%)	n (%)	n (%)		
	420 (100)	41 (9.8)	379 (90.2)		
**Gender**				Pearson Chi square	
Girls	207 (49.3)	19 (46.3)	188 (49.6)	0.158	*p* = 0.691
Boys	213 (50.7)	22 (53.7)	191 (50.4)		
**Gestational age**				Fisher	
<37weeks—Preterm	33 (8)	1 (2.6)	32 (8.6)	1.725	*p* = 0.346
>37 weeks	380 (92)	38 (97.4)	342 (91.4)		
**Birthweight**				Fisher	
<2500 g—Low Birth Weight	35 (9)	0.0	35 (9.8)	n/a	n/a
>2500 g	353 (91)	32 (100)	321 (90.2)		
**Breastfeeding**				Fisher	
Yes	382 (91.2)	40 (97.6)		2.306	*p* = 0.156
No	37 (8.8)	1 (2.4)			
**Duration of breastfeeding**				Mann–Whitney U	
Median in months (min–max)	10 (0–53)	18 (2–36)	8 (0–53)	3927.5	*p* = 0.000
**Family structure**				Pearson Chi square	
Both parents	216 (51.6)	19 (47.5)	197 (52.0)	2.506	*p* = 0.644
Both parents and others	57 (13.6)	6 (15)	51 (13.4)		
Single-parent families	70 (16.7)	5 (12.5)	65 (17.2)		
One parent and others	72 (17.1)	9 (22.5)	63 (16.6)		
Others	4 (1.0)	1 (2.5)	3 (0.8)		
**Childcare**				Pearson Chi square	
State pre-school	164 (39.1)	13 (31.7)	151 (40.0)	25.819	*p* = 0.000
Private pre-school	192 (45.8)	11 (26.8)	181 (47.9)		
Stays home w/mother	23 (5.5)	7 (17.1)	16 (4.2)		
Other	40 (9.5)	10 (24.4)	30 (7.9)		

**Table 3:** Sociodemographic characteristics of parents/caregivers.

	Immigrant Children	Children Born in Portugal to Portuguese Born Parents	Total	Statistical Test Value	*p* Value
Parents/caregivers	n (%)	n (%)	Total n (%)		
**Gender**				Pearson Chi square test	
Women	187 (86.5)	181 (89.8)	368 (87.6)	0.863	*p* = 0.353
Men	30 (13.8)	22 (10.2)	52 (12.4)		
**Age**				Mann–Whitney U test	
Median (min–max)	34 (20–75)	35 (18–69)	35 (18–75)	20017.000	*p* = 0.169
Parents’ educational level				Pearson Chi square test	
Lower education	41 (19.0)	27 (13.3)	68 (16.2)	6.563	*p* = 0.087
9 years completed	40 (18.5)	46 (22.7)	86 (20.5)		
Between 9 and 12 years	92 (42.6)	74 (36.5)	166 (39.6)		
University degree	43 (19.7)	56 (25.8)	90 (22.5)		
**Occupation**				Pearson Chi square test	
Intellectual and scientific jobs	19 (8.8)	36 (17.7)	55 (13.1)	51.944	*p* = 0.000
Mid-level technical professions	12 (5.5)	31 (15.3)	43 (10.2)		
Administrative jobs	12 (5.5)	29 (14.3)	41 (9.8)		
Personal and security services and sales	79 (36.4)	66 (32.5)	145 (34.5)		
Industry and construction qualified workers	7 (3.2)	6 (3.0)	13 (3.1)		
Non-qualified workers	75 (34.6)	21 (10.3)	96 (22.9)		
Other	13 (6.0)	14 (6.9)	27 (6.4)		
**Employment situation**				Pearson Chi square test	
Employed with a contract	137 (32.6)	158 (37.6)	295 (70.2)	14.400	*p* = 0.013
Employed without a contract	20 (4.8)	5 (2.5)	25 (6.0)		
Unemployed with benefits	10 (4.6)	6 (3.0)	16 (3.8)		
Unemployed without benefits	17 (7.8)	12 (5.9)	29 (6.9)		
Self-employed	16 (7.4)	9 (4.4)	25 (6.0)		
**Household monthly income**				Pearson Chi square test	
<500€	38 (18.5)	13 (6.7)	51(12.8)	33.052	*p* = 0.000
>500–750€	67 (32.7)	45 (23.3)	112 (28.1)		
>750–1000€	38 (18.5)	34 (17.6)	72 (18.1)		
>1000–1500€	37 (18.0)	43 (22.3)	80 (20.1)		
>1500–2000€	16 (7.8)	24 (12.4)	40 (10.1)		
>2000€	9 (4.4)	34 (17.6)	43 (10.8)		

**Table 4:** Strengths and Difficulties (SDQ) Scores: comparing immigrants with children born in Portugal to Portuguese parents.

Immigrant Status	1st Generation Immigrant Children	Children Born in Portugal	Statistical Test Value	*p* Value
	n (%)	n (%)		
Emotional problems score			Pearson Chi square	
Normal	31 (75.6)	268 (70.7)	3.039	*p* = 0.219
Borderline	2 (4.9)	54 (14.2)		
Abnormal	8 (19.5)	57 (15.1)		
Conduct problems score			Pearson Chi square	
Normal	14 (34.1)	207 (54.8)	6.480	*p* = 0.039
Borderline	11 (26.8)	76 (20.1)		
Abnormal	16 (39.0)	95 (25.1)		
Hyperactivity score			Pearson Chi square	
Normal	21 (51.2)	229 (60.6)	9.660	*p* = 0.008
Borderline	9 (22.0)	110 (29.1)		
Abnormal	11 (26.8)	39 (10.3)		
Peer problems score			Pearson Chi square	
Normal	29 (70.7)	284 (75.1)	1.286	*p* = 0.526
Borderline	5 (12.2)	52 (13.8)		
Abnormal	7 (13.0)	42 (11.1)		
Pro-social score			Pearson Chi square	
Normal	29 (70.7)	298 (79.3)	1.697	*p* = 0.369
Borderline	7 (17.1)	42 (11.2)		
Abnormal	5 (12.2)	36 (9.5)		
Total difficulties score			Pearson Chi square	
Normal	21 (51.2)	253 (66.9)	6.474	*p* = 0.039
Borderline	7 (17.1)	64 (16.9)		
Abnormal	13 (31.7)	61 (16.2)		

**Table 5:** Strengths and Difficulties Questionnaire (SDQ) Scores: comparing first generation immigrant children with children born in Portugal.

Immigrant Status	Immigrant Children	Children Born in Portugal to Portuguese Born Parents	Statistical Test Value	*p* Value
	n (%)	n (%)		
Emotional problems score			Pearson Chi square	
Normal	141 (65.0)	158(77.8)	12.014	*p* = 0.002
Borderline	30 (13.8)	26 (12.8)		
Abnormal	46 (21.2)	19 (9.4)		
Conduct problems score			Pearson Chi square	
Normal	113 (52.3)	108 (53.2)	1.183	*p* = 0.554
Borderline	49 (22.7)	38 (18.7)		
Abnormal	54 (25.0)	57 (28.1)		
Hyperactivity score			Pearson Chi square	
Normal	130 (60.2)	120 (59.1)	0.793	*p* = 0.673
Borderline	58 (26.9)	61 (30.0)		
Abnormal	28 (13.0)	22 (10.8)		
Peer problems score			Pearson Chi square	
Normal	156 (72.2)	157 (77.3)	1.461	*p* = 0.482
Borderline	32 (14.8)	25 (12.3)		
Abnormal	28 (10.3)	21 (10.3)		
Pro-social score			Pearson Chi square	
Normal	174 (80.9)	153 (75.7)	4.082	*p* = 0.130
Borderline	26 (12.1)	23 (11.4)		
Abnormal	15 (7.0)	26 (12.9)		
Total difficulties score			Pearson Chi square	
Normal	135 (62.5)	139 (68.5)	1.662	*p* = 0.436
Borderline	40 (18.5)	31 (15.3)		
Anormal	41 (19.0)	33 (16.3)		

**Table 6:** Factors associated to the chance of developing emotional and behavioral problems; immigrant status variable: immigrant children.

Model 1	aOR	95% CI	*p* Value
**Variables** **Gender of the child**			
Boy	1.122	0.723–1.743	0.607
Girl	reference		
**Gestational age**			
<37 weeks	2.546	0.975–6.645	0.056
≥37 weeks	reference		
**Immigrant Status**			
Child is immigrant	1.001	0.634–1.580	0.996
Child is not immigrant	reference		
**Household monthly income**			
<500 €	4.468	1.431–13.957	0.010
>500–750 €	2.283	0.812–6.419	0.118
>750–1000 €	2.243	0.784–6.419	0.132
>1000–1500 €	2.132	0.758–5.997	0.151
>1500–2000 €	1.612	0.495–5.257	0.428
>2000 €	reference		
**Parents Educational level**			
Lower education	2.491	1.115–5.564	0.026
9 years schooling	2.615	1.238–5.526	0.012
Between 9 and 12 years	1.908	0.988–3.682	0.054
University degree	reference		

**Table 7:** Factors associated with the chance of developing emotional and behavioral problems; immigrant status variable: 1st generation immigrant children.

Model 2	aOR	95% CI	*p* Value
**Variables** **Gender of the child**			
Boy	1.114	0.716–1.734	0.632
Girl	reference		
**Immigrant status**			
Child is 1st generation immigrant	2.247	1.062–4.756	0.034
Child is not 1st generation immigrant	reference		
**Household monthly income**			
<500 €	3.512	1.135–10.861	0.029
>500–750 €	1.968	0.705–492	0.196
>750–1000 €	1.947	0.682–5.562	0.213
>1000–1500 €	1.870	0.664–5.271	0.236
>1500–2000 €	1.531	0.469–5.001	0.480
>2000 €	reference		
**Parents Educational level**			
Lower education	2.995	1.303–6.884	0.010
9 years schooling	3.237	1.482–7.068	0.003
between 9 and 12 years	2.226	1.124–4.410	0.022
University degree	reference		

## Data Availability

The data presented in this study are available on request from the corresponding author.
